# Numerical Modeling of Anisotropic Particle Diffusion through a Cylindrical Channel

**DOI:** 10.3390/molecules29163795

**Published:** 2024-08-10

**Authors:** Michał Cieśla, Bartłomiej Dybiec, Monika Krasowska, Zuzanna Siwy, Anna Strzelewicz

**Affiliations:** 1 Institute of Theoretical Physics and Mark Kac Center for Complex Systems Research, Jagiellonian University, ul. St. Łojasiewicza 11, 30-348 Kraków, Poland; bartlomiej.dybiec@uj.edu.pl; 2 Faculty of Chemistry, Silesian University of Technology, Strzody 9, 44-100 Gliwice, Poland; monika.krasowska@polsl.pl (M.K.); anna.strzelewicz@polsl.pl (A.S.); 3 Department of Physics and Astronomy, University of California, Irvine, CA 92697, USA; zsiwy@uci.edu

**Keywords:** overdamped diffusion, channels and pores, first passage time, artificial pore, numerical modeling, stochastic dynamics

## Abstract

The transport of molecules and particles through single pores is the basis of biological processes, including DNA and protein sequencing. As individual objects pass through a pore, they cause a transient change in the current that can be correlated with the object size, surface charge, and even chemical properties. The majority of experiments and modeling have been performed with spherical objects, while much less is known about the transport characteristics of aspherical particles, which would act as a model system, for example, for proteins and bacteria. The transport kinetics of aspherical objects is an especially important, yet understudied, problem in nanopore analytics. Here, using the Wiener process, we present a simplified model of the diffusion of rod-shaped particles through a cylindrical pore, and apply it to understand the translation and rotation of the particles as they pass through the pore. Specifically, we analyze the influence of the particles’ geometrical characteristics on the effective diffusion type, the first passage time distribution, and the particles’ orientation in the pore. Our model shows that thicker particles pass through the channel slower than thinner ones, while their lengths do not affect the passage time. We also demonstrate that both spherical and rod-shaped particles undergo normal diffusion, and the first passage time distribution follows an exponential asymptotics. The model provides guidance on how the shape of the particle can be modified to achieve an optimal passage time.

## 1. Introduction

Advances in the field of ion channels in biological membranes, as well as nanopores in solid-state and polymer films, have led scientists to develop an interest in single-molecule and single-particle transport in nanoconfinement [1,2,3,4,5,6,7,8,9,10]. In the resistive pulse technique, the passage of single molecules or particles through a pore can be readily identified as a transient change in the transmembrane current, called the resistive pulse, whereby the amplitude and duration that can be linked to the object size and surface charge [11,12]. The approach provides a high throughput technique to analyze the suspensions of particles and molecules, including mixtures. The application of thin films and pores with a narrow constriction zone enabled extending the resistive pulse technique to DNA sequencing [13,14,15,16,17,18,19,20] and, more recently, protein sequencing [21,22,23], such that current blockage caused by individual nucleotides and amino acids can be identified and differentiated.

In the context of nanopore-based biosensors, there has been a significant focus on the regulation of the transit time of objects through the pore. On the one hand, the technique needs to offer a high throughput characterization of hundreds or even thousands of molecules or particles per second [24,25,26]. On the other hand, however, each molecule needs to spend a sufficient amount of time within the pore to enable detection and characterization. Yet, the majority of resistive pulse models have focused on spherical particles. In this study, we present a model system for the passage of aspherical and spherocylindrical objects, which can be applied to the analysis of specific types of proteins [27], bacteriophages [28], and bacteria [29,30], as well as synthetic particles used, e.g., in drug delivery [31].

A limited number of reports have described the passage of aspherical, rod-like particles through single pores. An early report by Golibersuch [32] provides analytical formulas that predict the magnitude of the current drop when ellipsoidal objects are in a pore. The passage of aspherical proteins through solid-state nanopores allowed researchers to ascertain both the protein shape and the dipole moment [27,33]. Resistive pulse experiments with polymer nanopores with an undulating opening diameter and silica rod-shaped particles tested the early predictions, and demonstrated significant rotations of the particles within the pore [34]. The passage of bacteria of various shapes through silicon nitride pores demonstrated how each type of bacterium could be distinguished based on its resistive-pulse characteristics [30]. Furthermore, the detection of gold nanorods in polymer and silicon nitride nanopores has been documented [35,36].

In this manuscript, we describe the kinetics of aspherical particles as they pass through a single cylindrical pore. We focus on the properties of diffusion as a function of particle size and shape in relation to the pore dimensions. The model we present describes the process of random walk performed by individual particles in a pore. Section 3 describes in detail the model we developed, together with its assumptions and the simplifications applied. This research is inspired by the experimental studies of diffusion [37], performed with submicron (400 nm in diameter) polystyrene particles passing through a single polymer micropore (11 μm in length and 0.7 μm in diameter). By balancing the forces acting on the particles, the authors were able to observe the particles’ random walk and determine their diffusion coefficient and electrokinetic velocity.

To describe the diffusion of particles in a pore, we use the Wiener process [38]. The methodology we present is closely connected to the concept of random walks [39,40], which describe the manner in which a particle forms its path from a multitude of random steps. The random walk approach has been used not only to explore diffusion processes [41,42], but also in the context of finance [43], search strategies [44,45], physiology [46], seismology [47], and numerous other systems. In the case of diffusion, the treatment based on the random walk models, along with appropriate extensions, allows for the distinction between normal, sub-, and superdiffusion when the movement is carried by a specific force [48,49] or through a crowded environment [50,51]. Therefore, although this approach is highly simplified, it offers significant insight into the transport properties of confined systems, which are of interest in this context.

**Figure 1:**
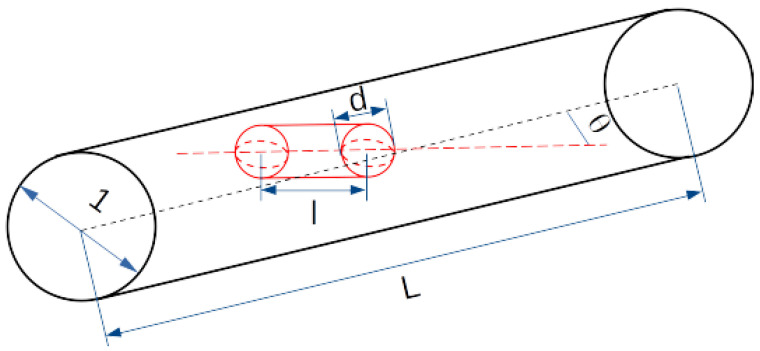
Scheme of a spherocylindrical particle within a cylindrical channel. The length of the channel is designated as *L*, and its width is of a unit length. The length of the cylindrical portion of the particle is *l*, resulting in a total length is l+d. The length-to-width ratio is f=(d+l)/d=1+l/d. The angle between the channel and particle axes is denoted by the variable θ.

In order to accurately describe the diffusion of anisotropic particles in a viscous medium, it is essential to consider the particle orientation in relation to the direction of motion [52,53]. Moreover, for particles that lack spherical symmetry, one must also consider the rotational degrees of freedom, which will play a significant role in the diffusive transport process. In the context of the aforementioned model, we consider the diffusive transport of single aspherical particles in a cylindrical pore. The nanopore has a diameter of one unit and a length of *L*. The particle is a spherocylinder of diameter *d* and length *l*, and is characterized by its length-to-width ratio of f=(l+d)/d=1+l/d (see Figure 1).

In order to simplify the model, it is common to numerically solve the Langevin equation of a traced particle using a set of effective, macroscopic parameters. For example, multiple collisions with the medium particles are modeled by a random force of specific variance and autocorrelation, whereas the energy dissipation is described by viscosity. Furthermore, these parameters can be combined into diffusion constants (see, e.g., Refs. [54,55,56,57]). Here, we employ a more simplified model, with the objective of developing a framework for simulating a random process that enables a molecule to traverse a channel. This approach allows for a significant reduction in computational time, provides a description that is independent of the microscopic details of the system, and allows for the identification of the fundamental physics of the process. In particular, our model considers the so-called overdamped limit, in which the subsequent positions and orientations of the particle can be described by the Wiener process [38]. This methodology has recently been justified both mathematically [58] and experimentally [59], and has been applied to other problems partially related to the diffusion of anisotropic particles [60].

## 2. Results and Discussion

### 2.1. Diffusion Type

The diffusion process through the channel was analyzed in terms of the mean squared displacement (see Appendix A):

(1) $$\langle {\left[\vec{x}\left(t\right)-\vec{x}\left(0\right)\right]}^{2}\rangle =\langle {\vec{x}}^{2}\left(t\right)\rangle =2D\times t^{\alpha },$$

where $\vec{x}\left(t\right)$ denotes the position of the particle’s center after *t* jumps [61] ($\vec{x}\left(0\right)=\left[0,0,0\right]$), *D* is the diffusion constant, and the exponent α determines the diffusion type (see Appendix A). Note that, for the normal diffusion, α=1, while for sub- or superdiffusion, α is smaller or greater than 1, respectively [41,62].

**Figure 2:**
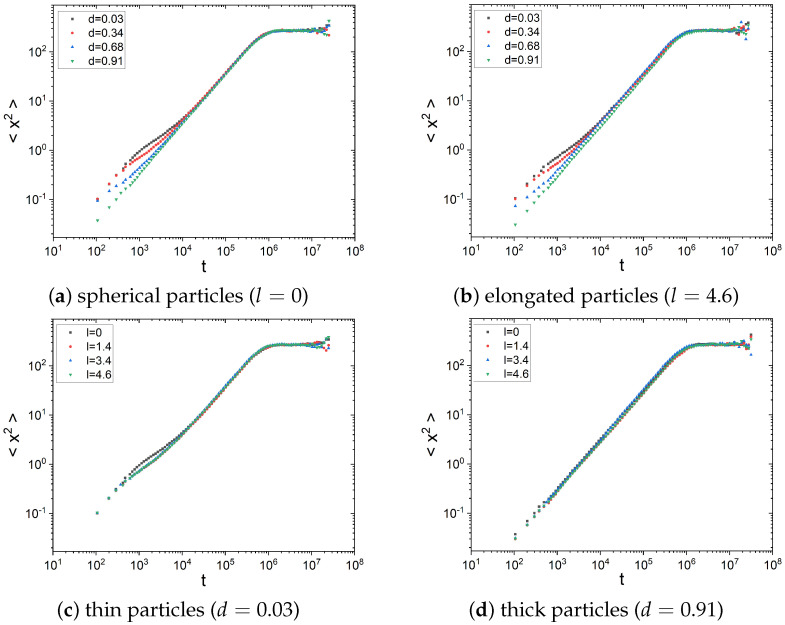
The mean squared displacement (MSD) $\langle {\vec{x}}^{2}\left(t\right)\rangle$ versus time for particles with different lengths and radii: (**a**) length l=0 (varying diameter), (**b**) length l=4.6 (varying diameter), (**c**) radius d=0.03 (varying length), (**d**) radius d=0.91 (varying length).

The dependence of $\langle {\vec{x}}^{2}\left(t\right)\rangle$ on time was recorded during each simulation, and the parameters *D* and α were determined. A few illustrative examples of the mean squared displacement (MSD) versus time are presented in Figure 2.

**Figure 3:**
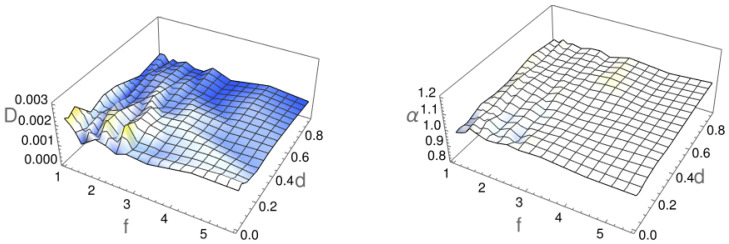
The diffusion constant *D* and the exponent α, which defines the diffusion type as a functions of the particle’s length-to-width ratio *f* and diameter *d*. The parameters *D* and α were extracted from fits of Equation (1) to the data obtained from numerical simulations.

As shown in Figure 2, the mean squared displacement obeys the scaling with time given by Equation (1), especially at intermediate times. Moreover, as the particle diameter *r* increases, the individual MSD curves become smoother (see Figure 2d) due to hindered rotation of thicker particles. The deviations from the scaling observed at the beginning and the end of the simulation are due to boundary conditions. To avoid the effects of reflecting and absorbing ends of the channel, the fitting of the MSD was performed for the time interval $\left[0.01t_{min},t_{min}\right]$, where $t_{min}$ denotes the minimal first passage time observed in the simulation of 5000 independent passing events. The fitted values of α and *D* are shown in Figure 3.

The diffusion constant *D* for different particle sizes was found to vary between 0.001 and 0.0024. In general, the diffusion coefficient is higher for smaller and less-elongated particles, which agrees with the intuition supported by the Stokes–Einstein–Smoluchowski–Sutherland relation [63,64,65], as well as previous observations [33,37,66]. The observed fluctuations and irregularities in Figure 3 are related to the fitting methodology. To confirm this, we conducted an additional experiment in which the number of independent trajectories that are averaged in Equation (1) was increased. This resulted in a notable reduction in the observed fluctuations and irregularities. On the other hand, the exponent α that defines the diffusion type for all particles was found to be very close to 1. This observation suggests that the particles perform normal diffusion, regardless of the particle length or thickness, which is in line with the findings reported in [59]. Our simulations, therefore, revealed that although the channel boundaries restrict particle motion by the introduction of trapping events, they do not slow down the transport sufficiently to change the normal diffusion into subdiffusion.

### 2.2. First Passage Time

Another important characteristic of the diffusive motion is the first passage time, defined as the shortest interval of time required for a particle to leave the pore for the first time. Its distribution is typically given by the exponential law, as follows:

(2) p(t)∝Aexp(−λt),

The distribution allowed us in turn to determine the survival probability S(t):

(3) $$S\left(t\right)=1-\int_{0}^{t}p\left(t\right)dt\propto \exp\left(-\lambda t\right).$$

**Figure 4:**
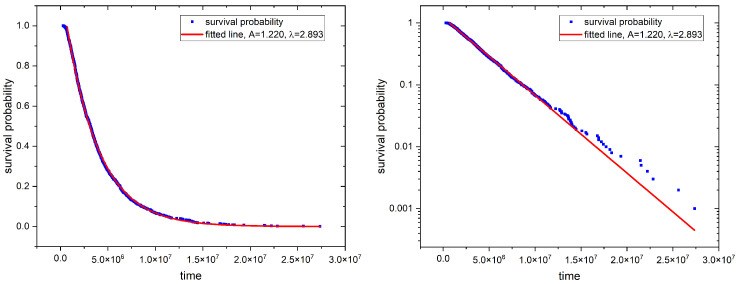
Sample survival probability for the particle of length-to-width ratio f=5.2 and the diameter d=0.34. Blue dots correspond to simulation data, while the red line is an exponential fit. The left panel is plotted in the lin–lin scale, while the log–lin scale is used for the right one.

The distribution S(t) quantifies the fraction of molecules that remain within the channel at a given time *t*. The exponent λ defines the rate of the probability decay, which reflects the diminishing probability of finding a tracer in a channel after a specified time *t*. We determined the first passage time for each particle, and the obtained distribution of survival probability is shown in Figure 4.

The recorded exponential tails (long-time asymptotics) indicate that this process is Markovian, i.e., the imposed geometrical constraints do not introduce memory to the system. Consequently, the next position and orientation of a particle depend only on its current state, regardless of earlier history, which is the case for the standard Wiener process in an empty space.

**Figure 5:**
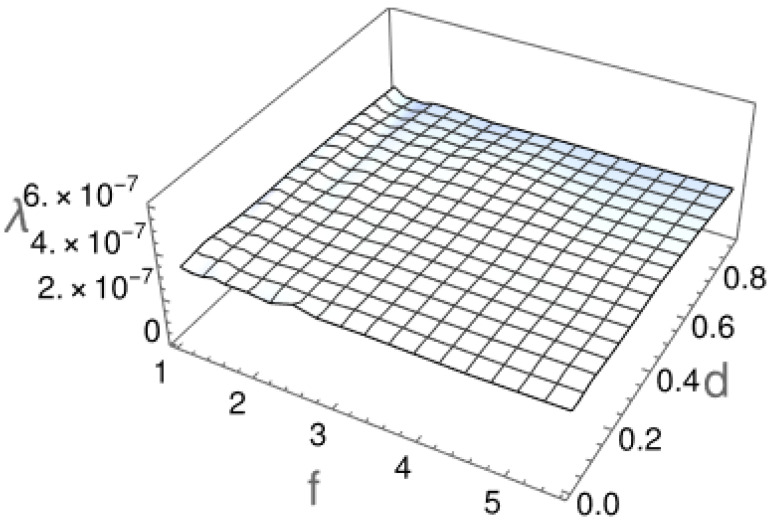
The exponent λ characterizing the decay of the survival probability (see Equation (3)) for particles of length-to-width ratio *f* and diameter *d*, based on 5000 independent simulations of a single particle diffusion.

Our simulations revealed that the parameter λ does not depend on the particle size (see Figure 5). This result supports the previous observation, based on the diffusion exponent α, that the character of the motion does not noticeably depend either on particle length or its thickness.

**Figure 6:**
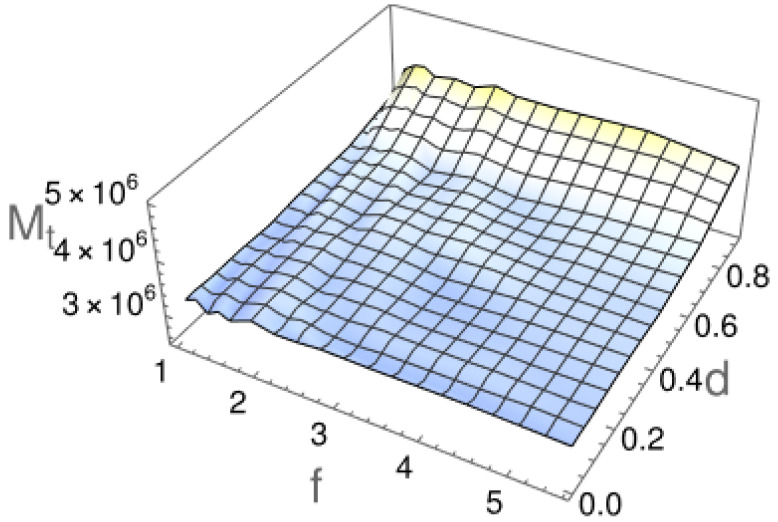
The Median of the first passage time as a function of the particle’s length and radius. The median was calculated from 5000 independent simulations of a spherocylindrical particle of length-to-width ratio *f* and diameter *d* passing through the channel.

Conversely, the median of the first passage time shown in Figure 6 indicates a slowdown for thicker particles, despite the almost identical tails of the survival probability for differently shaped particles. This is evident in Figure 5 and Figure 6. This apparent contradiction originates from the exponential tails present in S(t) only at long times. At short times t≈0, the survival probability exhibits different dependence, because a particle cannot leave the channel instantaneously. It is thus possible to determine a threshold time, after which an exponential decay of S(t) is visible. This time is directly related to the minimal time needed for a particle to pass through the channel. On the other hand, the equivalent information is carried by the median of passing times $M_{t}$, which is a physical quantity characterizing the transport process (see Figure 6).

Interestingly, there is no additional dependence of the median time $M_{t}$ on the particle length *l*. It is important to highlight that this phenomenon is not caused by considering rotational and translational movements separately. We checked the scenario in which both translation and rotation were considered simultaneously at each step and rejected if only one of them or both lead to a collision with the channel walls. The obtained results did not show significant differences from the results presented here.

This section showed that normal Markovian diffusion was observed for all particles we considered. The diffusion coefficient is the largest for small spherical particles. At the same time, the first passage time was found to depend on particle thickness only, such that thinner objects pass through the pore faster regardless of their elongation.

### 2.3. Angle Distribution

**Figure 7:**
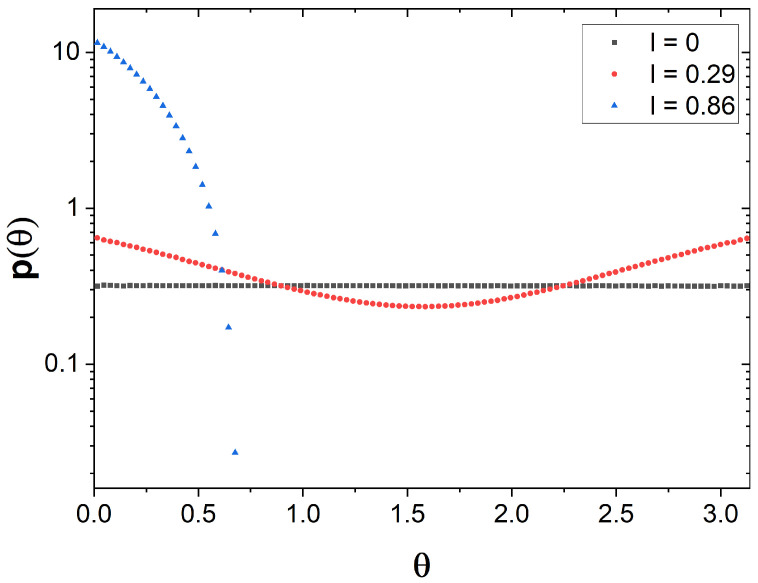
Histogram showing the distribution of particle orientation, i.e., the angle θ between the particle axis and the channel axis, for different molecule’s lengths (l+d=0.45,0.74,1.31, which correspond to f=1,1.6,2.9) with the fixed diameter d=0.45.

The last parameter we analyzed is the particle orientation at the exit time, θ, determined with respect to the long axis of the channel. Taking into account the specific angle distribution on a sphere (see details in Appendix B) the obtained distribution of θ (see Figure 7) confirm that all orientations of a spherical particle (f=1) occur at equal probabilities. As the aspect ratio of the particle increases, the distribution p(θ) becomes non-uniform. As expected, the longest particles we considered align nearly parallel to the channel axis.

To further characterize the angle distribution, we analyze its second moment

(4) $$\sigma ={\left(\frac{1}{\pi }\int_{0}^{\pi }\theta^{2}p\left(\theta \right)d\theta \right)}^{\frac{1}{2}}.$$

**Figure 8:**
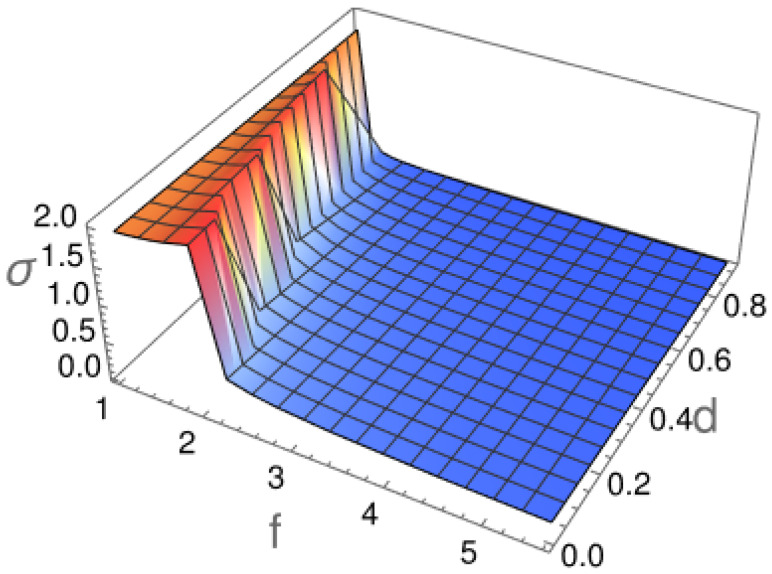
Parameter σ characterizing the distribution of particle orientation for different length-to-width ratios *f* and diameters *d*.

In the case of a uniform distribution, the standard deviation is given by $\sigma =\sqrt{\frac{\pi^{2}}{3}}\approx 1.814$. The measured values of σ for different particles are presented in Figure 8.

Here, as expected, we see a rapid decrease in σ with the increase in the particle length, reflecting the decaying ability of a particle to rotate in the restricted geometry of the channel.

## 3. Materials and Methods

We assume that the particle’s translations $\Delta \vec{x}$ and rotations $\Delta \varphi_{\hat{A}}$ are selected randomly. Here, $\hat{A}=\vec{A}/\left|\vec{A}\right|$ denotes the unit vector in the direction of $\vec{A}$ that represents the axis of rotation. The particle’s position and orientation are updated step-by-step according to (5) if and only if the update does not lead to the intersection of the particle’s surface with the channel’s boundaries

(5) $$\left\{\begin{array}{ccc} \Delta \vec{x} & = & \sigma_{x}\;\vec{\xi },\\ \Delta \varphi_{\hat{A}} & = & \sigma_{\varphi }\left(\hat{A}\right)\;\eta . \end{array}\right.$$

In Equation (5), $\sigma_{x}$ and $\sigma_{\varphi }\left(\hat{A}\right)$ denote a set of positive, scalar parameters characterizing random motion. These parameters are controlled by temperature, which measures the strength of random movements, and viscosity, which dissipates the kinetic energy. The components of the displacement vector $\Delta \vec{x}$ and the scalar η that determines the rotation angle are sampled from the normal distribution with a mean value of zero and unit variance. For the translational motion, we assume the same diffusion coefficient in the direction along the particle axis and in the direction that is perpendicular to the axis. The model can also be generalized to take into account possible differences in *D* in the two directions [52,53]. It is important to note that in the described model, particle rotation and translation properties are directly connected with the distribution of random variables, $\vec{\xi }$ and η. Thus, the observed diffusion coefficient reflects the width of these distributions and does not depend on particle size or mass, as long as the used distribution is constant. Note that this assumption would not be valid in the case of diffusion in unbounded physical systems. In the case described here, however, the effective diffusion is affected by the geometric constraints because the particle cannot cross the boundary of the pore walls. Consequently, this approach provides understanding of how diffusion properties depend on the relative sizes between the pore and the particles, which we study here.

Specifically, as the particle length increases, its motion becomes restricted due to the geometric confinement provided by the pore. This confinement effectively limits the particle’s ability to move across the pore unless its axis is oriented almost parallel to the channel axis. The direction of the rotation axis $\hat{A}$ ($\left|\hat{A}\right|=1$) is also selected randomly and uniformly on the two-dimensional sphere [58]. The rotation axis is assumed to pass through the geometric origin of the particle. The value of the parameter $\sigma_{\varphi }\left(\hat{A}\right)$ was adjusted to satisfy the energy equipartition law, i.e., the energy is uniformly distributed among all degrees of freedom. Thus, we use

(6) $$\sigma_{\varphi }\left(\vec{A}\right)=\sigma_{x}{\left({\hat{A}}^{T}M\hat{A}\right)}^{-\frac{1}{2}},$$

where ${\hat{A}}^{T}M\hat{A}$ is the moment of inertia around axis $\hat{A}$ (see [67] and Appendix C). In Equation (6), M is the inertia tensor of the spherocylinder (r=d/2):

(7) $$M=\left(\begin{array}{ccc} M_{xx} & 0 & 0\\ 0 & M_{yy} & 0\\ 0 & 0 & M_{zz} \end{array}\right),$$

where

(8) $$\begin{array}{ccc} M_{xx}=M_{yy} & = & m_{1}\left(\frac{1}{12}l^{2}+\frac{1}{4}r^{2}\right)+2m_{2}\left(\frac{2}{5}r^{2}+\frac{1}{4}l^{2}+\frac{3}{8}lr\right), \end{array}$$

and

(9) $$\begin{array}{ccc} M_{zz} & = & \left(\frac{1}{2}m_{1}+\frac{4}{5}m_{2}\right)r^{2}. \end{array}$$

The *z*-axis is parallel to the spherocylinder axis, while the *x* and *y* axes are perpendicular to it. *x*, *y* and *z*-axes cross at the geometric center of the spherocylinder. $m_{1}$ and $m_{2}$ are masses of the cylinder and the single hemisphere, respectively. We assume the particles have uniform density, thus masses $m_{1}$ and $m_{2}$ are related to the characteristic dimensions of the spherocylinder

(10) $$\frac{m_{1}}{m_{2}}=\frac{3l}{2r}.$$

The position of the geometric center of the particle is described using Cartesian coordinates, wherein the *z*-axis is parallel to the pore axis, as previously mentioned. The pore entrance is located at z=0, while the exit is situated at z=L. Initially, the particle is aligned along the pore axis with its center at $\vec{x}=\left[0,0,0\right]$. The simulation of each particle ends when the particle exits the channel, i.e., when z⩾L. In the pore, the particle is free to move in any direction, as long as its center remains between z=0 and z=L. The particle is, however, allowed to leave the channel only through z=L. In order to ensure that a spherocylinder does not leave the channel via the entry at z=0, the noise-induced displacement leading to the negative value of the *z* coordinate is rejected. Therefore, there is a reflecting boundary at z=0, and an absorbing boundary at z=L.

The random walk of each particle within the pore is modeled according to the following steps:

- Draw a random translation vector with coordinates selected from independent $N(0,\sigma_{x})$ distributions;
- Update the particle position until it does not lead to a collision with channel boundaries, or leaving the particle through the pore entry (z<0);
- Select a random axis of rotation;
- Calculate the particle’s moment of inertia with respect to rotations around the selected axis and the parameter $\sigma_{\varphi }$;
- Draw random angle from the $N(0,\sigma_{\varphi })$ distribution;
- Update the particle orientation if it does not lead to a collision with channel boundaries;
- Increase time;
- Verify if the particle is still within the channel;
- If a particle reaches the exit end of the channel (z>L), record the first passage time (stopping time) and finish the simulation. Otherwise, return to the first step.

In our simulation, the time unit is defined as the number of random steps, wherein each step consists of one translation and one rotation. Note that the number of steps also includes the rejected jumps.

Our approach of observing subsequent steps and rotations allows us to determine the type of diffusion a given particle follows. During a single simulation, we record the particle position from which the mean squared displacement (MSD) is calculated as a function of the number of steps. We also measure the number of steps a particle needs to leave the channel, the first passage time, and the histogram of the particle orientations inside the channel.

To obtain satisfactory statistics for each specific particle size and channel size, we performed 5000 independent numerical simulations. The parameter $\sigma_{x}$ was set to 0.05, which was selected to optimize the time required for a single simulation while ensuring sufficient sampling of traced parameters. In the simulations, the values of length-to-width ratios are chosen to match the experimental conditions [34]. The channel’s length was set to 14.3, and the width played the role of the unit length. The particle dimensions vary between 1⩽f⩽5.6 and 0.03⩽d⩽0.9. The limiting case of f=1 (l=0) corresponds to a spherical particle, because the rod connecting two hemispheres is absent in that situation (c.f. Figure 1).

## 4. Conclusions

The numerical simulations of particles in a channel permitted a systematic examination of the impact of particle shape on diffusion processes within a restricted geometry. The motion of the particles was modeled by the Wiener process, accounting for both the translational and the rotational degrees of freedom. Our analysis demonstrated that all particles that we considered, irrespective of their shape, followed normal diffusion. This finding indicates that the confinement of the particles and their interactions with the channel walls in our system were not able to transform the normal diffusion into subdiffusion. However, the diffusion constant is sensitive to the particle size and shape such that smaller and less elongated molecules are characterized by larger diffusion coefficients. We also found that thicker molecules are characterized by a larger median of the first passage time compared to thinner particles. Surprisingly, however, other diffusion properties, such as the decay of the survival probability, were found the same for all particles. This result might seems counter-intuitive, but it is consistent with the constraints that we imposed in the model. Namely, the same model of random motion was applied to all particles. Finally, as expected, the rotation of longer particles was found to be limited by the channel boundaries. Consequently, the distribution of the direction of the particles as they pass through the channel depends strongly on the particle size.

The numerical study of the noise-driven motion of anisotropic particles in a channel provides a more comprehensive understanding of the interrelationships between the macroscopic properties of the observed diffusive motion and the microscopic dynamics of individual particles. The ability to determine the influence of particle shape on the diffusion within a pore will be beneficial in the analysis of experimental data from biological and artificial nanopores, as well as in the optimization of particle shape for sensing applications. Further studies on the role of shape in the diffusion of single molecules, e.g., viruses, particles and cells, could boost the development of the resistive pulse technique for the identification of aspherical objects from mixtures. The model we have developed could be further modified to account for anomalous diffusion observed in some systems [68,69,70]. Modifications would include other distributions or random displacements, such as Cauchy or other heavy-tailed distributions, external forces or flows, or the inclusion of obstacles [49]. 

## Data Availability

The numerical data are available at: https://doi.org/10.57903/UJ/QRHLMI. More information will be provided upon reasonable request.

## Appendix A. Normal and Anomalous Diffusion

The trajectory x(t) of Brownian motion can be described in terms of the following stochastic process:

(A1) $$\frac{dx}{dt}=\xi \left(t\right),$$

where ξ(t) is the Gaussian white noise (〈ξ(t)〉=0 and 〈ξ(t)ξ(s)〉=2Dδ(t−s)). The formal solution of Equation (A1) is

(A2) $$x\left(t\right)=\int_{0}^{t}\xi \left(s\right)ds,$$

where, without loss of generality, we assumed x(0)=0. The solution x(t) is given by the Wiener process [38]. From Equation (A2), one can calculate

(A3) $$\begin{array}{ccc} \langle x^{2}\left(t\right)\rangle & = & \langle \int_{0}^{t}\int_{0}^{t}\xi \left(u\right)\xi \left(v\right)dudv\rangle \\ & = & \int_{0}^{t}\int_{0}^{t}\langle \xi (u)\xi (v)\rangle dudv\\ & = & \int_{0}^{t}\int_{0}^{t}2D\delta \left(u-v\right)\rangle dudv\\ & = & \int_{0}^{t}2Ddu\\ & = & 2Dt \end{array}$$

from which it can be seen that $\langle x^{2}\left(t\right)\rangle \propto t^{1}$. For x(0)=0 the $\langle x^{2}\left(t\right)\rangle$ is equivalent to the mean squared displacement. Equation (A3) shows that for Brownian motion, the mean squared displacement scales linearly in time. Equation (A3) can be generalized into higher number of dimensions

(A4) $$\langle {\left[\vec{x}\left(t\right)-\vec{x}\left(0\right)\right]}^{2}\rangle =2D\times t.$$

For the sake of simplicity, we have omitted the additional multiplicative constant that would otherwise be required due to the increased number of dimensions.

The Brownian motion is a classic example of the so-called normal diffusion. It is known, however, that the MSD does not always scale linearly with time [71,72,73]. Therefore, we can assume that

(A5) $$\langle {\left[\vec{x}\left(t\right)-\vec{x}\left(0\right)\right]}^{2}\rangle =2D\times t^{\alpha },$$

where α is the exponent defining the diffusion type. The reference case of α=1 corresponds to the normal diffusion, while α<1 represents subdiffusion. Finally, α>1 stands for superdiffusion. These names indicate whether the MSD scales with time faster (superdiffusion) or slower (subdiffusion) than in the case of normal diffusion. Subdiffusion is typically produced by trapping events, whereas superdiffusion is due to anomalously long jumps [41,42,73].

## Appendix B. Renormalization of the Angle Distribution

Let us assume that we measure the angle θ between a vector pointing from the center of the unit sphere to a random (uniformly distributed) point on the sphere and the channel axis. Points on the sphere are selected from the uniform distribution on the unit sphere

(A6) $$\tilde{p}\left(\theta ,\varphi \right)=\frac{1}{4\pi },$$

where θ (0⩽θ⩽π) is the polar angle, i.e., the angle between the particle axis and the channel axis when the molecule leaves the channel. φ (0⩽φ⩽2π) stands for the azimuthal angle, thus the angle between the orthogonal projection of the rotation axis onto the plane perpendicular to the particle axis and a fixed direction on that plane. The $\tilde{p}\left(\theta \right)$ distribution can be calculated as

(A7) $$\tilde{p}\left(\theta \right)=\int_{0}^{2\pi }p\left(\theta ,\varphi \right)\sin\varphi d\varphi =\frac{1}{2}\sin\theta .$$

The density of $\tilde{p}\left(\theta \right)$, as seen in Equation (A7), is non-uniform. The histogram H(θ) of the angle θ can be re-normalized as

(A8) $$p\left(\theta \right)=\frac{H(\theta )}{\int_{bb}^{eb}p\left(\theta \right)d\theta }=\frac{2H(\theta )}{\cos(bb)-\cos(eb)},$$

where bb and eb indicate the beginning and end of the bin containing the angle θ and H(θ) is the probability, calculated from the histogram, of observing a given value of θ. The re-normalized distribution p(θ) (for a spherical molecule) is uniform (see Figure 7).

## Appendix C. Energy of Rotating (Rigid) Molecule

A moving, rigid particle’s kinetic energy *K* consists of two components: the translational motion of the center of mass ($K_{T}$) and the rotation along the center of mass ($K_{R}$). We will focus first on the rotational part only.

(A9) $$K_{R}=\frac{1}{2}{\vec{\omega }}^{T}M\vec{\omega },$$

where $\vec{\omega }=\left[\omega_{xx},\omega_{yy},\omega_{zz}\right]$ and M is the tensor of inertia. Within the studied model, instead of generating three angles, we select a random axis of rotation $\vec{A}$, and the particle is then rotated about this axis. Consequently, the angular velocity $\vec{\omega }=\dot{\varphi }\hat{A}$ (where $\hat{A}$ is the unit vector indicating the rotation axis) must be transformed to the principal axes (see Equations (7)–(9)). In the principal axes basis,

(A10) $${\vec{\omega }}^{T}=\dot{\varphi }\left[\hat{A}\cdot \hat{x},\hat{A}\cdot \hat{y},\hat{A}\cdot \hat{z}\right]=\dot{\varphi }\left[{\hat{A}}_{x},{\hat{A}}_{y},{\hat{A}}_{z}\right]$$

and the rotational energy reads

(A11) $$K_{R}=\frac{1}{2}{\dot{\varphi }}^{2}\left[{\hat{A}}_{x},{\hat{A}}_{y},{\hat{A}}_{z}\right]M{\left[{\hat{A}}_{x},{\hat{A}}_{y},{\hat{A}}_{z}\right]}^{T}=\frac{1}{2}{\dot{\varphi }}^{2}{\hat{A}}^{T}M\hat{A},$$

showing that ${\hat{A}}^{T}M\hat{A}$ can be interpreted as the inertia moment along the $\hat{A}$ axis.

The kinetic energy of the translational motion is given as:

(A12) $$K_{T}=\frac{1}{2}m{\vec{v}}^{T}\vec{v},$$

where $\vec{v}=v\hat{v}$ is the particle velocity. Here, $v=|\vec{v}|$ and $\hat{v}=\vec{v}/v$. The energy equipartition $\langle K_{T}\rangle =\langle K_{R}\rangle$ leads to

(A13) $$\langle {\dot{\varphi }}^{2}\rangle {\hat{A}}^{T}M\hat{A}=m\langle v^{2}\rangle .$$

Therefore, the energy equipartition establishes a relationship between $\langle {\dot{\varphi }}^{2}\rangle$ and $\langle v^{2}\rangle$, and the model parameters $\sigma_{x}$ and $\sigma_{\phi }\left(\hat{A}\right)$ are linked by Equation (6).
